# The role of a transparent cap in the endoscopic removal of foreign bodies in the esophagus: A propensity score‐matched analysis

**DOI:** 10.1111/1751-2980.12833

**Published:** 2019-12-25

**Authors:** Rui Fang, Bin Cao, Qian Zhang, Peng Li, Shu Tian Zhang

**Affiliations:** ^1^ Department of Gastroenterology Beijing Friendship Hospital, Capital Medical University Beijing China; ^2^ National Clinical Research Center for Digestive Diseases Beijing China; ^3^ Beijing Digestive Disease Center Beijing China; ^4^ Beijing Key Laboratory for Precancerous Lesion of Digestive Diseases Beijing China; ^5^ Clinical Epidemiology and Evidence‐Based Medicine Unit Beijing Friendship Hospital, Capital Medical University Beijing China

**Keywords:** cap, endoscopy, esophagus, foreign bodies, upper gastrointestinal tract

## Abstract

**Objective:**

To investigate the effectiveness and safety of transparent cap‐assisted endoscopy in removing foreign bodies in the esophagus.

**Methods:**

Patients with foreign body lodged in the esophagus who received a transparent cap‐assisted or conventional endoscopy between October 2004 and July 2018 were retrospectively enrolled. Propensity score matching was performed. The success rate of the endoscopic procedure, procedure time, clearness of endoscopic view and adverse event rate were compared between the two groups.

**Results:**

Of the 838 patients who had a foreign body lodged in the esophagus, 728 (86.9%) underwent endoscopic intervention. After matched by prospensity score, 224 patients each received either transparent cap‐assisted endoscopy or conventional endoscopy. No difference was noted between the two groups in terms of the success rate (100% vs 99.1%, *P* = 0.499). Transparent cap‐assisted endoscopy was associated with shorter procedure time for removing jujube pits ([4.24 ± 2.81] min vs [7.62 ± 8.15] min, *P* = 0.001), fish bones ([2.99 ± 2.15] min vs [6.49 ± 6.54] min, *P* < 0.001) and other sharp objects ([4.29 ± 3.36] min vs [10.60 ± 19.79 min], *P* = 0.027) and higher rates of clear endoscopic views in extracting jujube pits, fish bones, poultry bones and other sharp objects (98% vs 43.4%, 97.5% vs 74.1%, 100% vs 81.3% and 100% vs 82.7%; all *P* < 0.05). No significant differences in the rates of adverse event were observed between the groups (*P* = 1.000).

**Conclusion:**

Transparent cap‐assisted endoscopic technique is effective and safe for removing sharp foreign bodies in the esophagus.

## 1 INTRODUCTION

Ingestion of a foreign body, including long, sharp object and short, blunt object, as well as food bolus, in the upper gastrointestinal (GI) tract is a common clinical emergency.^1^ Most ingested objects are located in the esophagus, accounting for up to 86.9% of the cases,^2^, ^3^, ^4^, ^5^ and those at this location have the highest adverse event rate.^6^ Although most (80%‐90%) foreign bodies can pass through spontaneously without a need for any intervention, some may even lead to severe complications, including aspiration, hemorrhage and perforation. Complications caused by foreign body ingestion, especially sharp‐pointed objects, have been reported to occur in 15% to 35% of the cases.^1^, ^2^ Thanks to the development of endoscopic techniques, foreign bodies can now be treated with emergency endoscopic intervention.^7^, ^8^ Complete obstruction caused by sharp‐pointed foreign bodies, batteries, and food bolus impaction that enter the esophagus requires emergency endoscopic intervention within 2‐6 hours. Other foreign bodies lodged in the esophagus can be treated by urgent endoscopic removal within 24 hours.^9^ Some studies even reported an endoscopic intervention rate of as high as 76%.^10^, ^11^ Because of the narrow lumen of the esophagus, traditional endoscopic removal can be challenging, and a clear visual field is essential for the procedure. Recently, transparent cap‐assisted techniques have been proposed to be used during the endoscopic removal of a foreign body.^12^, ^13^ A few studies have suggested that transparent cap‐assisted endoscopy could be used to manage foreign bodies in the esophagus, including food boluses, which shortened the procedure time, cleared the visual field, and reduced the rate of adverse event compared with conventional endoscopic techniques.^14^, ^15^ So far, few reports have focused on transparent cap‐assisted endoscopic removal of a foreign body, including all types of true foreign bodies and food boluses, from the esophagus; therefore, we conducted a propensity score‐matched study to investigate the effectiveness and safety of transparent cap‐assisted endoscopy, and compare with those of conventional endoscopy in the removal of foreign bodies lodged in the esophagus.

## 2 PATIENTS AND METHODS

### 2.1 Patients

This retrospective cohort study was conducted at the Beijing Friendship Hospital, Capital Medical University (Beijing, China). Medical records of all consecutive patients who had ingested a foreign body in the esophagus as confirmed by a barium contrast examination, chest radiography or computed tomography (CT) between October 2004 and July 2018 were screened. Patients aged <18 years, pregnant women, those with foreign bodies located in other part of the GI tract other than the esophagus, those whose foreign bodies passed through spontaneously and needed no interventions, or those who had undergone surgical intervention were excluded from the study. All patients provided their written informed consent to undergo the endoscopic procedure beforehand. All the procedures were performed in accordance with the ethical standards of the Institutional Ethical Committee of Beijing Friendship Hospital, and the National Research Committee, and was conducted according to the Declaration of Helsinki (Brazil, 2013). Written informed consent was waived due to the retrospective study design.

### 2.2 Endoscopic removal of foreign bodies

Removal of foreign bodies was performed by several experienced endoscopists (each has performed more than 2000 endoscopies) using an Olympus endoscope (GIF‐Q260J or GIF‐H260; Olympus, Tokyo, Japan) under topical pharyngeal anesthesia by lidocaine hydrochloride mucilage. The patients were assigned to receive conventional endoscopy, which used a traditional endoscope to perform the procedure, or a transparent cap‐assisted endoscopy, as determined by experienced endoscopists based on the patient's medical history, clinical results, and the type and location of foreign body. Transparent cap‐assisted endoscopy involved the use of a disposable distal‐wide‐opening oblique transparent cap (disposable distal attachment, model D‐206‐05; Olympus) of an outer diameter of 18.1 mm, which was fixed on the tip of the endoscope with sticky tape. Accessories (all from Olympus), including grasping forceps, polypectomy snares, baskets, retrieval nets and latex rubber hoods, were used during the procedure according to the size and shape of the foreign body. If conventional techniques failed to remove the foreign body, the transparent cap‐assisted technique was used by the endoscopist when the patient was physically stable. In cases where there was a high risk of severe adverse events such as airway compromise, penetration of the esophagus, aortic or tracheal fistulae or cardiac tamponade, as evaluated by the endoscopists, surgical intervention was then recommended to the patient. After endoscopic removal of the foreign body all patients immediately underwent a complete esophagogastroduodenoscopy to assess the adverse events and potential lesions in the esophagus, such as bleeding, perforation, mucosal tear or pathological disease. Conservative treatment, argon plasma coagulation, GI decompression and clipping were performed according to the patient's condition when complications occurred. The foreign body was measured with a caliper and the measurements were recorded by assistants after the procedure.

### 2.3 Data collection and outcomes

Two researchers collected the data of all cases independently. Patients’ characteristics included their gender and age, and the type, length and location of the foreign body were recorded. Data of the type of accessories used, any underlying esophageal diseases and their history of esophageal diseases were also recorded. The outcomes, including the success rate of the procedure, the procedure time, the clearness of the endoscopic view, procedure‐related adverse events (bleeding, perforation and mucosal tear, etc) and risk factors of prolonged procedure time, were compared between the two groups.

### 2.4 Definitions

The procedure time was calculated from the initiation of the endoscopic removal to the time point that complete removal was achieved, but did not include the time taken to assess upper GI diseases, adverse events and additional interventions after the removal of the foreign body.

**Figure 1:**
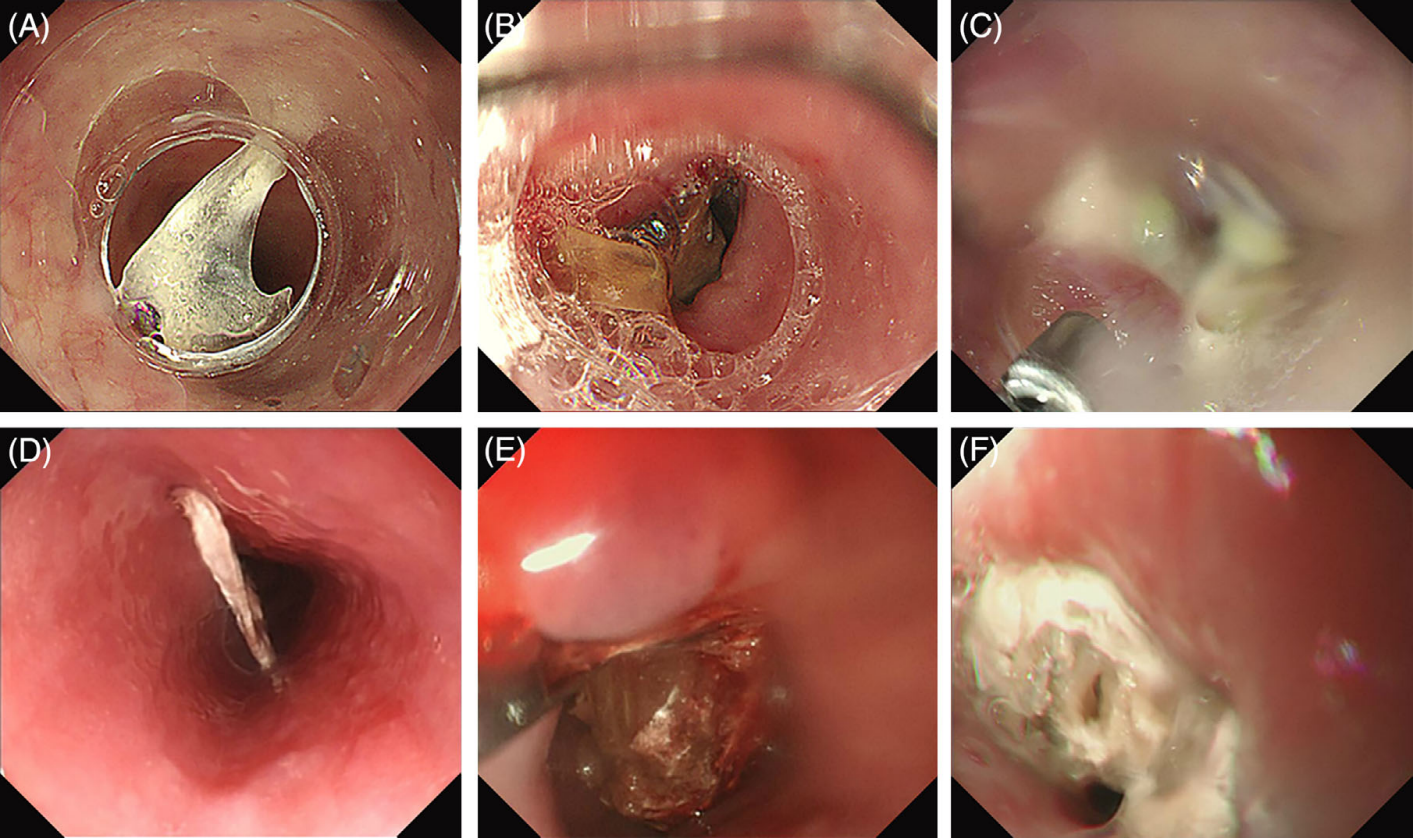
Illustration of the clearness of the endoscopic view as evaluated by the reviewers. Transparent cap‐assisted endoscopy: A, grade A; B, grade B; C, grade C. Conventional endoscopy: D, grade A; E, grade B; F, grade C

To our knowledge, there has been no consensus on the classification of the clearness of endoscopic view. Therefore, in the current study the clearness of endoscopic view was evaluated by two reviewers separately based on the same endoscopic images, and was classified into three grades based on previous studies and the reviewer's experiences (Figure 1). Grade A indicates that the endoscopic visual field is clear enough to evaluate completely the shape and position of the foreign body and the conditions of the esophageal mucosa. In grade B the shape of the foreign body can be observed but its position and mucosal conditions can be evaluated only partially. If the foreign body cannot be visualized clearly due to esophageal contraction, bleeding or other conditions, the grade of clearness of the endoscopic view is classified as grade C. Any disagreement in classification was resolved by discussion with a third reviewer.

Benign esophageal strictures included anastomotic stricture, achalasia, peptic ulcer, varices, hiatus hernia and reflux esophagitis, and so on. Adverse events included bleeding, perforation and mucosal tear.

### 2.5 Statistical analysis

Categorical variables were presented as numbers and percentages, whereas continuous variables were presented as mean ± standard deviation. Categorical variables were evaluated using the χ^2^ test or Fisher's exact test, when appropriate. Continuous variables were first analyzed for normal distribution using SPSS 22.0 (IBM, Armonk, NY, USA). A Student's *t*‐test was performed for continuous variables with a normal distribution. To balance the potential confounding variables at baseline, a propensity score method was used to match the patients’ gender and age, and by the MatchIt R package^16^ as well as the nearest neighbor matching method (ratio = 1:1) for the location and length of the foreign body. Each patient had a distance measure as the score of propensity. Patients in the transparent cap‐assisted endoscopy group who did not have a counterpart regarding the distance measure among patients in the conventional group were excluded from the propensity score matching analysis. After the matching process the risk factors for prolonged procedure time were estimated by odds ratio (OR) and 95% confidence interval (CI) using univariate and multivariate logistic regression analysis.

Propensity score matching was performed by R software version 3.5.1 (The R Foundation, Vienna, Austria). Other data were analyzed by SPSS software version 22.0 (IBM). A two‐sided *P* value < 0.05 was considered statistically significant.

## 3 RESULTS

### 3.1 Clinical characteristics of patients with esophageal foreign bodies

**Figure 2:**
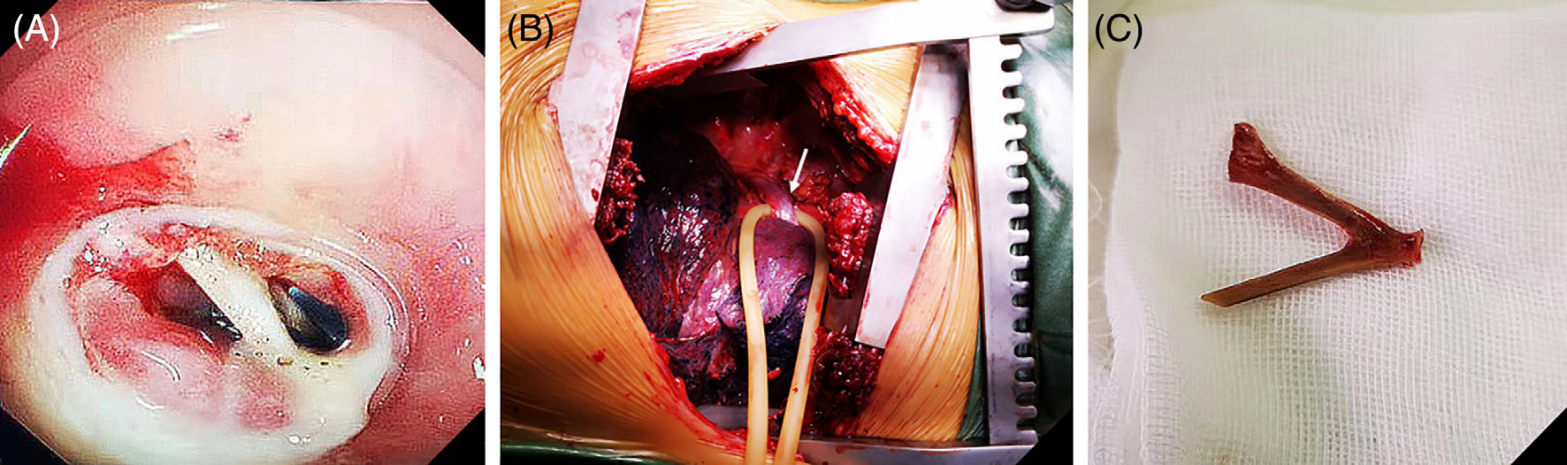
Surgery for foreign body removal. A, under transparent cap‐assisted endoscopy, a bone‐like foreign body penetrated into the wall of the esophagus with local mucosal erosion and purulent secretion. B, a foreign body was found in the proximal esophagus (arrow) during thoracotomy. C, a V‐shaped chicken bone was removed

**Figure 3:**
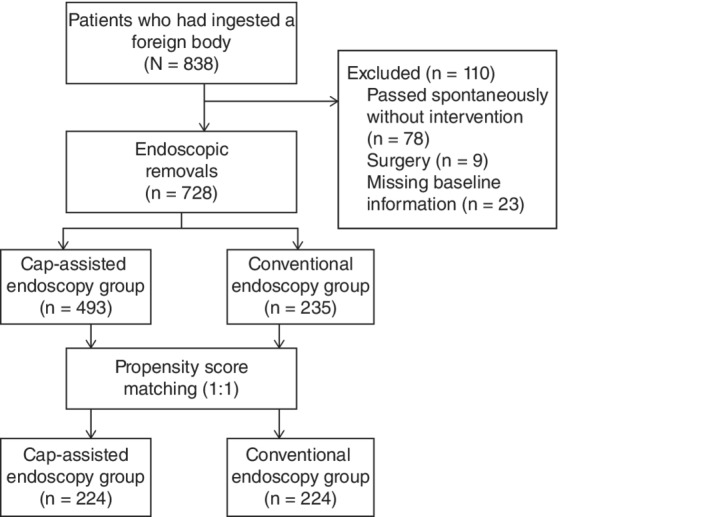
Flowchart of the study

A total of 838 patients with confirmed foreign body ingestion in the esophagus between October 2004 and July 2018 were screened for eligibility. Of these patients, foreign body passed out of the esophagus spontaneously without endoscopic intervention in 78 patients, and nine patients were assigned for emergency surgery because of a high risk of adverse events. An example of surgical intervention for removal of the foreign body is shown in Figure 2. Another 23 patients were also excluded due to missing baseline information. Finally, 728 patients underwent endoscopic removal. Among them, 493 patients were treated by using a cap‐assisted endoscopy and the other 235 underwent a conventional endoscopy (Figure 3). The patients’ characteristics are summarized in Table 1. After propensity score matching, 224 pairs of patients in the two groups were matched in their baseline characteristics, including patient's gender and age, as well as the location and length of the foreign body.

**Table 1:** Patients’ characteristics before and after propensity score matching

	Before matching (N = 728)			After matching (N = 448)		
	Cap‐assisted endoscopy (n = 493)	Conventional endoscopy (n = 235)	*P* value	Cap‐assisted endoscopy (n = 224)	Conventional endoscopy (n = 224)	*P* value
Gender, n (%)			0.001			0.569
Male	213 (43.2)	132 (56.2)		121 (54.0)	127 (56.7)	
Female	280 (56.8)	103 (43.8)		103 (46.0)	97 (43.3)	
Age, y (mean ± SD)	60.9 ± 16.9	62.1 ± 18.5	0.347	62.8 ± 16.7	62.4 ± 18.2	0.297
Location of foreign body in the esophagus, n (%)			0.000			0.918
Proximal	381 (77.3)	134 (57.0)		133 (59.4)	129 (57.6)	
Middle	88 (17.8)	74 (31.5)		67 (29.9)	69 (30.8)	
Distal	24 (4.9)	27 (11.5)		24 (10.7)	26 (11.6)	
Length of foreign body (mm), n (%)			0.021			0.869
≤20	141 (28.6)	67 (28.5)		60 (26.8)	56 (25.0)	
21‐30	271 (55.0)	110 (46.8)		110 (49.1)	110 (49.1)	
>30	81 (16.4)	58 (24.7)		54 (24.1)	58 (25.9)	

Abbreviation: SD, standard deviation

### 3.2 Types of foreign bodies, underlying GI diseases of the patients and accessories used during endoscopic procedures between the two groups after propensity score matching

Types of foreign bodies, underlying GI diseases of the patients and the accessories used during endoscopic procedures were compared between the two groups after propensity score matching, as shown in Table 2. The most common type of foreign body was a jujube pit (34.6%), followed by bones (28.6%), other sharp objects (20.1%) and a food bolus (16.7%), respectively. Other sharp objects ingested included tablets with packaging, dental bridgework, keys and iron wire. The types of foreign bodies ingested differed significantly between the two groups (*P* < 0.001), with more jujube pit and fish bones treated by the transparent cap‐assisted endoscopy, while poultry bones, food bolus and other sharp objects were more commonly treated by using the conventional endoscopy.

**Table 2:** Comparison of types of foreign bodies, underlying gastrointestinal (GI) diseases of the patients and accessories used during the endoscopic procedure between the transparent cap‐assisted endoscopy group and the conventional endoscopy group

	After matching (N = 448)		
	Cap‐assisted endoscopy (n = 224)	Conventional endoscopy(n = 224)	*P* value
Types, n (%)			<0.001
Jujube pit	102 (45.5)	53 (23.7)	
Fish bones	40 (17.9)	27 (12.0)	
Poultry bones	29 (12.9)	32 (14.3)	
Food bolus	15 (6.7)	60 (26.8)	
Other sharp objects	38 (17.0)	52 (23.2)	
Underlying GI diseases, n (%)			<0.001
None	209 (93.3)	164 (73.2)	
Benign stricture	9 (4.0)	36 (16.1)	
Malignant stricture	2 (0.9)	13 (5.8)	
Other benign diseases	4 (1.8)	11 (4.9)	
Accessories, n (%)			<0.001
Not used	3 (1.3)	2 (0.9)	
One kind used	209 (93.3)	175 (78.1)	
Two kinds used	11 (4.9)	43 (19.2)	
Three or more used	1 (0.4)	4 (1.8)	

There was a significant difference in concomitant GI diseases between the two groups (*P* < 0.001). Altogether 66.7% of the cases with a food bolus obstruction were associated with benign or malignant esophageal stricture (data not shown).

As for accessories used during endoscopic removal, the cap‐assisted endoscopy group was more likely to use one type of or no device than the conventional endoscopy group (94.6% vs 79.0%, *P* < 0.001), while the conventional endoscopy group more commonly used two or more types of accessories (5.3% vs 21.0%, *P* < 0.001).

### 3.3 Clinical outcomes of the patients after propensity score matching

Clinical outcomes of the patients in the subgroup analysis after propensity score matching are presented in Table 3. There was no difference between the transparent cap‐assisted endoscopy group and the conventional endoscopy group in terms of the success rate of the procedure (100% vs 99.1%, *P* = 0.499). Two patients underwent cap‐assisted endoscopy after a failed conventional endoscopy and both had their foreign bodies removed successfully.

**Table 3:** Clinical outcomes in the subgroup analysis after propensity score matching

Types of foreign body	Jujube pit (n = 155)			Fish bones (n = 67)			Poultry bones (n = 61)			Other sharp objects (n = 90)			Food bolus (n = 75)		
	Cap‐assisted endoscopy (n = 102)	Conventional endoscopy (n = 53)	*P* value	Cap‐assisted endoscopy (n = 40)	Conventional endoscopy (n = 27)	*P* value	Cap‐assisted endoscopy (n = 29)	Conventional endoscopy (n = 32)	*P* value	Cap‐assisted endoscopy (n = 38)	Conventional endoscopy (n = 52)	*P* value	Cap‐assisted endoscopy (n = 15)	Conventional endoscopy al (n = 60)	*P* value
Procedure time, min (mean ± SD)	4.24 ± 2.81	7.62 ± 8.15	0.001	2.99 ± 2.15	6.49 ± 6.54	0.001	3.20 ± 2.60	7.86 ± 15.92	0.067	4.29 ± 3.36	10.60 ± 19.79	0.027	6.63 ± 7.28	6.83 ± 6.50	0.900
Clearness of endoscopic view, n (%)			0.000			0.014			0.049			0.026			0.247
Grade A	100 (98.0)	23 (43.4)		39 (97.5)	20 (74.1)		29 (100)	26 (81.2)		38 (100)	43 (82.7)		15 (100)	55 (91.7)	
Grade B	2 (2.0)	22 (41.5)		1 (2.5)	5 (18.5)		0 (0)	4 (12.5)		0 (0)	6 (11.5)		0 (0)	5 (8.3)	
Grade C	0 (0)	8 (15.1)		0 (0)	2 (7.4)		0 (0)	2 (6.3)		0 (0)	3 (5.8)		0 (0)	0 (0)	
Adverse events, n (%)	10 (9.8)	6 (11.3)	0.768	3 (7.5)	3 (11.1)	0.612	2 (6.9)	2 (6.3)	0.919	1 (2.6)	5 (9.6)	0.190	0 (0)	0 (0)	
Bleeding	2 (2.0)	3 (5.7)	0.216	1 (2.5)	2 (7.4)	0.341	1 (3.4)	1 (3.1)	0.944	1 (2.6)	1 (1.9)	0.822	0 (0)	0 (0)	
Conservative treatment	0 (0)	0 (0)		0 (0)	0 (0)		1 (3.4)	0 (0)		0 (0)	0 (0)		0 (0)	0 (0)	
APC	0 (0)	0 (0)		0 (0)	1 (3.7)		0 (0)	0 (0)		0 (0)	0 (0)		0 (0)	0 (0)	
Hemostatic clipping	2 (2.0)	3 (5.7)		1 (2.5)	1 (3.7)		0 (0)	1 (3.1)		1 (2.6)	1 (1.9)		0 (0)	0 (0)	
Perforation	7 (6.9)	3 (5.7)	0.773	2 (5.0)	1 (3.7)	0.801	0 (0)	1 (3.1)	0.337	0 (0)	3 (5.8)	0.132	0 (0)	0 (0)	
Conservative treatment	4 (3.9)	2 (3.8)		1 (2.5)	0 (0)		0 (0)	0 (0)		0 (0)	0 (0)		0 (0)	0 (0)	
Decompression	2 (2.0)	0 (0)		1 (2.5)	1 (3.7)		0 (0)	1 (3.1)		0 (0)	0 (0)		0 (0)	0 (0)	
Clipping	1 (1.0)	1 (1.9)		0 (0)	0 (0)		0 (0)	0 (0)		0 (0)	3 (5.8)		0 (0)	0 (0)	
Mucosal tear	2 (2.0)	1 (1.9)	0.975	0 (0)	0 (0)		1 (3.4)	0 (0)	0.290	0 (0)	1 (1.9)	0.390	0 (0)	0 (0)	

Abbreviation: APC, argon plasma coagulation; SD, standard deviation

Sung et al^17^ reported that the type of foreign bodies ingested influenced the risk of complications, which further affects procedure time and the clearness of the endoscopic view. Therefore, we further analyzed patient's clinical outcomes according to the type of foreign body. Use of transparent cap‐assisted endoscopy was associated with a significantly shorter procedure time compared with conventional endoscopy for removing jujube pits, fish bones and other sharp objects from the esophagus ([4.24 ± 2.81] min vs [7.62 ± 8.15] min, *P* = 0.001; [2.99 ± 2.15] min vs [6.49 ± 6.54] min, *P* = 0.001; [4.29 ± 3.36] min vs [10.60 ± 19.79] min, *P* = 0.027, respectively), whereas the procedure time for retrieving poultry bones and food boluses were comparable between the two groups (*P* = 0.067 and 0.900, respectively).

Grade A endoscopic view for extracting jujube pits, fish bones, poultry bones and other sharp objects were significantly more commonly achieved in the cap‐assisted endoscopy group than in the conventional endoscopy group (98.0% vs 43.4%, *P* < 0.001; 97.5% vs 74.1%, *P* = 0.014; 100% vs 81.2%, *P* = 0.049; and 100% vs 82.7%, *P* = 0.026, respectively). There was no significant difference in the clearness of the endoscopic view between the two groups during the removal of food bolus (*P* = 0.247).

The overall rates of adverse events, including bleeding, perforation or mucosal tear in the two groups were both 7.1% (16/224) (*P* = 1.000), and the rates of bleeding, perforation and mucosal tear were all comparable between the two groups during the removal of all types of foreign bodies (all *P* > 0.05). There was no requirement of surgery or death after removing the foreign body.

### 3.4 Risk factors for prolonged procedure time

As the average procedure time for the entire cohort was 6.02 minutes, we defined a prolonged procedure time as more than 6 minutes, and a short procedure time as 6 minutes or less. After adjustments, the multivariate analysis showed that the cap‐assisted endoscopic technique protected against a prolonged procedure time (OR 0.35, 95% CI 0.22‐0.54, *P* < 0.001). The patients’ age was associated with the procedure time (age 46‐65 y: OR 2.19, 95% CI 1.08‐4.40, *P* = 0.029; age over 65 y: OR 2.35, 95% CI 1.21‐4.55, *P* = 0.011; Table 4).

**Table 4:** Risk factors for prolonged procedure time (>6 minutes) by logistic regression analysis

	Case, n (%)	Crude OR (95% CI)	*P* value	Adjusted OR (95% CI)	*P* value
Gender					
Male (n = 248)	68 (27.4)	1.00			
Female (n = 200)	61 (30.5)	1.16 (0.77‐1.75)	0.474		
Age					
≤45 y (n = 82)	14 (17.1)	1.00		1.00	
46‐65 y (n = 142)	43 (30.3)	2.11 (1.07‐4.15)	0.031	2.19 (1.08‐4.40)	0.029
>65 y (n = 224)	72 (32.1)	2.30 (1.21‐4.36)	0.011	2.35 (1.21‐4.55)	0.011
Location of foreign body in the esophagus					
Proximal (n = 262)	74 (28.2)	1.00			
Middle (n = 136)	35 (25.7)	0.88 (0.55‐1.41)	0.595		
Distal (n = 50)	20 (40.0)	1.69 (0.91‐3.17)	0.099		
Length of foreign body					
≤20 mm (n = 116)	31 (26.7)	1.00		1.00	
21‐30 mm (n = 220)	53 (24.1)	0.87 (0.52‐1.46)	0.596	0.84 (0.49‐1.44)	0.525
>30 mm (n = 112)	45 (40.2)	1.84 (1.05‐3.22)	0.032	1.76 (0.98‐3.15)	0.056
Types of foreign body					
Fish bones (n = 67)	12 (17.9)	1.00			
Jujube pits (n = 155)	46 (29.7)	1.93 (0.95‐3.95)	0.070		
Poultry bones (n = 61)	11 (18.0)	1.01 (0.41‐2.49)	0.986		
Food bolus (n = 75)	26 (34.7)	2.43 (1.11‐5.33)	0.026		
Other sharp objects (n = 90)	34 (37.8)	2.78 (1.31‐5.93)	0.008		
Endoscopy used					
Conventional endoscopy (n = 224)	87 (38.8)	1.00		1.00	
Cap‐assisted endoscopy (n = 224)	42 (18.8)	0.36 (0.24‐0.56)	0.000	0.35 (0.22‐0.54)	0.000

Abbreviations: CI, confidence interval; OR, odds ratio

## 4 DISCUSSION

An ingested foreign body lodged in the esophagus is one of the most common situations requiring emergency endoscopic intervention in clinical practice, and can cause severe adverse events, and even death. Common foreign bodies ingested include sharp‐pointed objects, short blunt objects and food boluses.^1^ Traditional endoscopic technique for removing a foreign body in the esophagus requires a flexible endoscopy with accessories, with a high success rate, a low incidence of adverse events and an additional advantage of detecting potential GI diseases during the procedure.^1^, ^9^ With the development of endoscopic techniques, transparent caps have been widely used to assist endoscopic treatments, including endoscopic mucosal resection and submucosal dissection. Recently, as an additional technique, a transparent cap‐assisted endoscopy has been found to be associated with a shorter operation time, higher success rate and clearer visual field compared with conventional endoscopy in removing a foreign body.^14^ However, these studies were limited by their focus on the location and type of the foreign body. For example, Zhang et al^15^ focused on the upper esophagus and a study by Ooi et al^14^ involved only food boluses. Therefore, studies focusing on various types of foreign bodies located in the esophagus are needed.

In the present study, we found that the most common foreign body in the esophagus was a jujube pit, followed by bones. Food boluses were relatively rare, accounting for only 16.7% of ingested objects. The most common types of foreign bodies reported are different between the West countries and China, as food boluses are more commonly reported in the West populations, while bones are more likely to be found in Chinese patients.^4^, ^5^, ^9^, ^18^, ^19^ This difference may be due to the various eating habits; in the West world meat is usually cooked and eaten off the bones, while in China meat is more likely to be cooked with bones. Additionally, there are dietary differences in north and south China. People in the north China tend to enjoy jujubes, while those in the south prefer poultry and fish. Previous articles are mostly on people from southern China.

This study showed endoscopists who performed the transparent cap‐assisted endoscopy mostly used none or only one type of device than the conventional endoscopy group. This finding has not been reported previously. This might be related to the broad lumen and improved vision provided by the transparent cap so that the shape of the foreign body and its position in the esophageal lumen can be clearly and comprehensively observed, which contributes not only to the accurate selection of auxiliary devices but also to the ease of reaching the foreign body and performance in the esophagus using these devices. As a result, a transparent cap can be used to avoid repeated replacement of devices and shorten the operation time. Additionally, the difference in clinical expense between the two groups is worth further research in a prospective study.

During the removal of sharp foreign bodies, it was obvious that transparent cap‐assisted endoscopy had the advantage of a shorter procedure time and a clearer endoscopic view over conventional endoscopy, and the logistic regression analysis in our study showed that the cap‐assisted technique was a protective factor for prolonged procedure time. These results were similar to those of Zhang et al’ study.^15^ When sticking to the wall of the esophagus, sharp objects can cause congestion, edema or bleeding of the esophageal mucosa and even spasmodic contractions, blurring the lens of the endoscope and seriously affecting the endoscopic field of vision. With a transparent cap on the tip of the lens, endoscopic device can visualize the esophageal lumen effectively, keep a clear endoscopic vision field, and facilitate the capture of sharp objects, which shortens the procedure time. We also found that patients’ age was a risk factor for prolonged procedure time. These risk factors may be associated with more comorbidities in older patients who should be treated with caution.

Regarding food boluses, Ooi et al^14^ suggested that a transparent cap‐assisted endoscopy could shorten operation time and reduce the incidence of adverse events in *en bloc* resection compared with a traditional endoscopy. However, the present study showed that there was no significant difference between the two techniques in terms of procedure time and the rate of adverse events, as well as the clearness of the endoscopic view. This may be due to pathological stenosis of the esophagus. Our study found that food bolus obstructions were more commonly associated with benign and malignant esophageal strictures, and the incidence of stricture was higher in our patients than that in other studies,^2^, ^14^, ^20^, ^21^ which may be related to the high morbidity of esophageal cancer in China.^22^ After a food bolus obstruction caused by stricture has occurred, esophageal lumen above the impaction is usually expanded, providing a wider field of view for endoscopic removal and making the operation easier. In these circumstances, the utility of a transparent cap is correspondingly reduced.

In this study, the incidence of adverse events was comparable between the cap‐assisted endoscopy group and the conventional endoscopy group. While mucosal injury during the removal of foreign bodies was less reported in the transparent cap‐assisted group in Zhang et al’ study.^15^ The reason might be that all mucosal tears in our study were local mucosal injuries caused by foreign bodies other than procedure‐related mucosal injury. During the retrieval of sharp items it was easier to capture objects and protect the mucosa from laceration with a transparent cap on the tip of the endoscope. Consequently, our results showed that cap‐assisted endoscopy is safe for removing foreign bodies from the esophagus.

This study had several strengths. First, our study might have included the largest sample size during a time period of 14 years, showing that the transparent cap is effective for the removal of foreign bodies in the esophagus. Furthermore, to our knowledge, no previous reports have focused on the removal of a foreign body in the esophagus using transparent cap‐assisted endoscopy, involving different types of foreign bodies such as bones and other sharp objects. In addition, the propensity score matching method was used to balance the baseline variables and minimize potential confounding effects, making our results reliable.

This study also had some limitations. First, because this was a single‐center retrospective study there might have had selection bias. Although we performed precise propensity score matching to minimize the bias and balance confounding variables in order to make the results comparable, same inevitable selection bias still existed as other retrospective studies. Moreover, due to the lack of video resources, the clearness of the endoscopic view could be evaluated only by still images, and there have been no recognized criteria for performing the evaluation. Based on previous data,^23^ we defined the degree of the endoscopic view as precise as possible, made sure each case meet the standard of endoscopic images, and ensured that all judgments were agreed by endoscopy experts.

In conclusion, transparent cap‐assisted endoscopy was associated with a shorter procedure time and a clearer endoscopic view than conventional endoscopy and it is as safe as the traditional technique. Thus, transparent cap‐assisted endoscopy may be considered a superior method of removing sharp foreign bodies lodged in the esophagus. Large prospective randomized controlled trials are needed to further confirm these findings.
